# Protection Efficacy of Oral Bait Probiotic Vaccine Constitutively Expressing Tetravalent Toxoids against *Clostridium perfringens* Exotoxins in Livestock (Rabbits)

**DOI:** 10.3390/vaccines8010017

**Published:** 2020-01-08

**Authors:** Jing Bai, Xinyuan Qiao, Yingying Ma, Meijing Han, Shuo Jia, Xinning Huang, Bing Han, Li Wang, Yijing Li, Yigang Xu

**Affiliations:** 1 Heilongjiang Key Laboratory for Animal Disease Control and Pharmaceutical Development, College of Veterinary Medicine, Northeast Agricultural University, Harbin 150030, China; baijing@westlake.edu.cn (J.B.); qiaoxinyuan@neau.edu.cn (X.Q.); myy930823@163.com (Y.M.); h18246186256@163.com (M.H.); jiashuo0508@163.com (S.J.); 15945682816@163.com (X.H.); h55627197@163.com (B.H.); wanglicau@163.com (L.W.); yijingli@163.com (Y.L.); 2 School of Life Sciences, Westlake University, Hangzhou 310024, China; 3 Northeast Science Inspection Station, Key Laboratory of Animal Pathogen Biology of Ministry of Agriculture of China, Harbin 150030, China

**Keywords:** *Clostridium perfringens*, exotoxins, toxoids, bait probiotic vaccine, immune protection

## Abstract

*Clostridium perfringens* is an opportunistic pathogen. Its main virulence factors are exotoxins, which are the etiological agents of enteritis necroticans and enterotoxemia caused in livestock (cattle, sheep, and rabbits). Here, we demonstrated effective immune protection for rabbits against α, β, and ε exotoxins of *C. perfringens* provided by an oral tetravalent bait probiotic vaccine delivering α, ε, β1, and β2 toxoids of *C. perfringens*. Results showed that the recombinant probiotic had good segregational stability and good colonization ability in the rabbit intestinal tract. Oral administration of the probiotic vaccine can effectively elicit significant levels of antigen-specific mucosa sIgA and sera IgG antibodies with exotoxin-neutralizing activity. Additionally, oral immunization with the probiotic vaccine effectively promoted lymphoproliferation and Th1/Th2-associated cytokine production. The protection rate of immunized rabbits with the probiotic vaccine was 80% after challenging rabbits with a combination of *C*. *perfringens* (toxinotypes A, C, and D) and exotoxin mixture, which was better than the 60% provided by a commercial inactivated *C. perfringens* A, C, and D trivalent vaccine. Moreover, obvious histopathological changes were observed in the intestinal tissues of rabbits in the commercial vaccine and PBS groups. The bait probiotic vaccine can provide effective protection against *C. perfringens* exotoxins, suggesting a promising *C. perfringens* vaccination strategy.

## 1. Introduction

*Clostridium perfringens* is an opportunistic pathogen that is commonly found in the gastrointestinal tract of livestock and poultry. It is a Gram-positive, anaerobic, rod-shaped, and spore-forming bacterium that is the major causative agent of enteritis necroticans and enterotoxemia in animals. It can also cause food poisoning in humans [1,2,3]. The main virulence factors of *C*. *perfringens* are its exotoxins including at least 12 toxins. Although some researchers recently proposed that *C. perfringens* toxinotypes should be expanded to G (A to G seven toxinotypes) [4], the traditional *C. perfringens* toxinotyping scheme still recognizes that *C*. *perfringens* is classified into five toxinotypes (A, B, C, D, and E) according to the exotoxins α, β, ε, and ι [5]. Among these exotoxins, α, β, and ε are the most important pathogenic factors of *C. perfringens*. α-toxin is excreted by all *C. perfringens* toxinotypes, and it is also the only exotoxin produced by toxinotype A. α-toxin has phospholipase and sphingomyelinase activities, and it can cause hemolysis, tissue necrosis, edema, and other effects [6,7]. β-toxin, also known as β1-toxin, is a key lethal virulence factor mainly produced by *C. perfringens* toxinotype B and C, which has strong neurotoxicity, lethality, and necrotic effects. It can cause fatal hemorrhagic enterocolitis and enterotoxemia [8]. β2-toxin, which has similar biological activities to β1-toxin, can cause gastrointestinal diseases [9]. ε-toxin, which is produced by *C. perfringens* toxinotypes B and D, is a potent pore-forming toxin that can cause central nervous system diseases in animals [10]. Since *C. perfringens* produces many exotoxins, particularly α, β, and ε exotoxins, which are the main virulence factors of the pathogenic bacterium, a multivalent vaccine would be more effective against *C. perfringens* exotoxins.

Generally, the use of antibiotics like virginiamycin and tylosin is very common in livestock husbandry to prevent *C. perfringens* infection. Although in-feed antibiotics effectively control bacterial diseases, the abuse of antibiotics has brought unavoidable negative effects on the environment and human health. In particular, antibiotic resistance and its persistence in the environment are causes of growing worldwide concern. Moreover, vaccines like injected killed vaccines can effectively prevent *C. perfringens* infection [11]. For example, in China, *C. perfringens* (type A) inactivated vaccine for rabbits, *C. perfringens* (types A and C) bivalent inactivated vaccine for piglets, and *C. perfringens* (types A, B, C, D) tetravalent inactivated vaccine for sheep (Qilu Animal Health Products Co., LTD., Jinan, China) are available on the market. However, the vaccines often fail to resist the effects of exotoxins produced by *C. perfringens* in the circulatory system and intestinal tracts. *C. perfringens* is commonly found in intestinal tracts, and its exotoxins are mainly absorbed via the intestinal mucosa, causing disease development. Therefore, a vaccine that could induce efficacious protective immune responses against *C. perfringens* toxins in the circulatory system and in the intestinal tracts is a promising approach.

Oral vaccination may be a better choice because oral vaccine stimulation can effectively induce secretory immunoglobulin A (sIgA)-based antigen-specific mucosal immune response and IgG-based systemic immune response, providing effective host protection [12]. Furthermore, the antigen delivery carrier is essential for developing effective oral vaccines. An ideal delivery carrier can deliver antigens to intestinal mucosa to induce effective antigen-specific immune responses while also being safe and beneficial to the body. *Lactobacillus*, commonly found in food and intestinal tracts, is an important probiotic. Many *Lactobacillus* strains can promote adhesive interactions with intestinal epithelial cells [13], prevent epithelial cell barrier injury [14], ameliorate inflammation [15], modulate innate immunity [16], and regulate dendritic cell and T cell immunological functions [17,18]. Therefore, the use of *Lactobacillus* as an antigen delivery carrier to express heterologous antigens for oral vaccine development has attracted much attention in this field, including using *Lactobacillus* strains to express classical swine fever virus E2 protein [19], dendritic cells, or microfold cells and dendritic cells-targeting peptide fused with porcine epidemic diarrhea virus COE antigen [20,21], bovine viral diarrhea virus E2 protein [22], and α-toxoid of *C. perfringens* [23].

We previously constructed a genetically engineered *Lactobacillus* strain pPG-E-α-β2-ε-β1/*L. casei* constitutively expressing α, ε, β1, and β2 toxoids of *C. perfringens*, obtaining good immunogenicity in mice orally administered with the recombinant *Lactobacillus* strain [24]. However, it is not clear if the recombinant probiotic oral vaccine can effectively induce mucosal and systemic immune responses and provide effective immune protection for livestock against *C. perfringens* exotoxins. Here, we prepared a bait particle vaccine with the pPG-E-α-β2-ε-β1/*L. casei* (*Lactobacillus casei* was used as antigen delivery carrier to express α, ε, β1, and β2 toxoids of *C. perfringens*. Subsequently, the recombinant probiotic expressing the tetravalent toxoids was made into bait particles with feed raw materials, which was used as oral vaccine for immunization). We further evaluated the immunogenicity and immune-protective effect of the bait particle vaccine in rabbits against α, β, and ε natural exotoxins via oral vaccination.

## 2. Materials and Methods

### 2.1. Animal and Bacterial Strains

Eight-week-old healthy New Zealand White (NZW) rabbits purchased from Harbin Fenying Farming Company, China were kept under clean conventional conditions with free access to standard chow diet and water. *Lactobacillus casei* ATCC 393 (*L. casei*) and recombinant lactobacillus pPG-E-α-β2-ε-β1/*L. casei* constitutively expressing *C. perfringens* α, ε, β1, and β2 toxoids were constructed by our laboratory [24] and grown anaerobically in de Man, Rogosa, and Sharpe (MRS) broth (Sigma, St. Louis, MO, USA) at 37 °C without shaking. *C. perfringens* toxinotype A (C57-1), toxinotype C (CACC-61), and toxinotype D (CCVC-81) were purchased from the China Institute of Veterinary Drug Control, (Beijing, China). Animal experiments were carried out in accordance with the recommendations in the Guide for the Care and Use of Laboratory Animals of the National Institutes of Health. The protocol (NEAU2018024) was approved by the Ethical Committee for Animal Experiments of Northeast Agricultural University, China.

### 2.2. Constitutive Protein Expression

Here, enhanced green fluorescent protein (EGFP) was used as a marker to screen recombinant lactobacillus. So, we first identified the expression of EGFP without any specific inducers. Briefly, pPG-α-β2-ε-β1/*L. casei* was cultured in MRS broth at 37 °C for 16 h without shaking before centrifuging at 12,000× *g* for 10 min. After washing three times with sterile PBS, the cells were observed using ultra high-resolution microscopy. Subsequently, constitutive toxoid expression was identified using western blotting. Briefly, pPG-α-β2-ε-β1/*L. casei* was grown in basic MRS broth at 37 °C for 16 h using glucose, sucrose, galactose, lactose, raffinose, fructose, and trehalose as carbon sources, respectively. After centrifuging, the cell pellets were lysed with 2× sodium dodecyl sulfate (SDS) buffer before placing in a boiling water bath for 10 min and centrifuging. Next, the supernatant was analyzed using 10% SDS-polyacrylamide gel electrophoresis (SDS-PAGE) combined with western blotting. For western blotting, the mouse anti-α, β2, ε, and β1 toxoid monoclonal antibody that was prepared in our laboratory (to prepare these monoclonal antibodies, α, β2, ε, and β1 toxoid protein was respectively expressed by *E. coli* followed by purification, immunization, fusion of immunized mice splenocytes and SP2/0. After that, the monoclonal antibodies targeting each toxoid protein were screened followed by evaluation of the specificity) was used as the primary antibody, and horseradish peroxidase (HRP)-conjugated goat anti-mouse IgG (Sigma, Louis, MO, USA) was used as the secondary antibody.

### 2.3. Recombinant Plasmid pPG-α-β2-ε-β1 Segregational Stability

Segregational stability of recombinant plasmid pPG-α-β2-ε-β1 in *L. casei* was evaluated as previously described [12]. Briefly, pPG-α-β2-ε-β1/*L. casei* was cultured in MRS broth at 37 °C until optical density at 600 nm (OD_600_) of approximately 1.0. Subsequently, serial subcultures were performed by diluting the culture (1:100) in fresh broth and growing it until OD_600_ ≈ 1.0. To determine the structural stability of recombinant plasmid pPG-α-β2-ε-β1 after 50 generations, 100 μL of diluted culture from every five generations was plated onto MRS agar plate and cultured at 37 °C for 12 h. Ten colonies were selected at random and analyzed using colony PCR assay (the primer sequences were that forward—5′-GATACAGATAATAATTTCTCAAAGGATAAT-3′ and reverse—5′-AATAGCTGTTACTTTGTGAGTAAGCC-3′ with annealing at 50 °C. The expected PCR result size is 3468 bp) combined with gene sequencing to detect the integrity of the α-β2-ε-β1 gene in the plasmid.

### 2.4. Digestive Tract Tolerance and Adhesion Ability of Recombinant Lactobacillus

One-milliliter of pPG-α-β2-ε-β1/*L. casei* cultured in MRS broth (OD_600_ ≈ 1.0) was centrifuged, and the following experiments were performed. (1) Bile tolerance test: the cells were re-inoculated in 1 mL MRS broth supplemented with 0.05%, 0.1%, 0.2%, 0.3%, 0.4%, or 0.5% of bile (wt/vol), and continually cultured at 37 °C for 5 h. (2) Hypertonic environment tolerance test: The cells were re-inoculated in 1 mL MRS broth containing 4%, 5%, 6%, 7%, 8%, or 9% of NaCl, and continually cultured at 37 °C for 5 h. (3) Gastric environment tolerance test: the cells were re-inoculated in 1 mL of simulated gastric juice (1 mol/L hydrochloric acid and 1 g pepsin per 100 mL) with pH 1.5, 2.5, 3.5, or 4.5 and continually cultured at 37 °C for 3 h. (4) Intestinal fluid environment tolerance test: the cells were re-inoculated in 1 mL of simulated intestinal fluid (0.68 g KH_2_PO_4_ and 1 g trypsase per 100 mL) and continually cultured at 37 °C for 3 h. In each experiment, the number of live bacteria was counted at 30-min intervals using the plate method.

Adhesion ability of recombinant lactobacillus in the intestinal tracts of NZW rabbits was evaluated as previously described [12] with some modification. Briefly, eight-week-old NZW rabbits (*n* = 18) were orally administered approximately 10^11^ CFU/mL of cFDA-SE-labeled pPG-α-β2-ε-β1/*L. casei*. Three NZW rabbits were randomly selected and sacrificed on days 1, 4, 7, 11, 15, and 20 after oral administration. Colon, jejunum, and ileum were extracted from each rabbit. Individual sections were cut longitudinally, and visible residual food particles or fecal material was removed from the intestinal tracts. Subsequently, the intestinal mucosa was scraped and placed in sterile PBS. After full oscillation, the microbes were dislodged from the mucosa tissue, fixed with formaldehyde (0.75%, *v*/*v*), and analyzed using flow cytometry. Normal rabbits (*n* = 6) and rabbits treated with pPG-α-β2-ε-β1/*L. casei* (unlabeled recombinant lactobacillus) (*n* = 6) were used as negative control. The results were calculated as the following formula: the value of flow cytometry (experimental group)—the value of flow cytometry (negative control group).

### 2.5. Immunization

**Figure 1:**
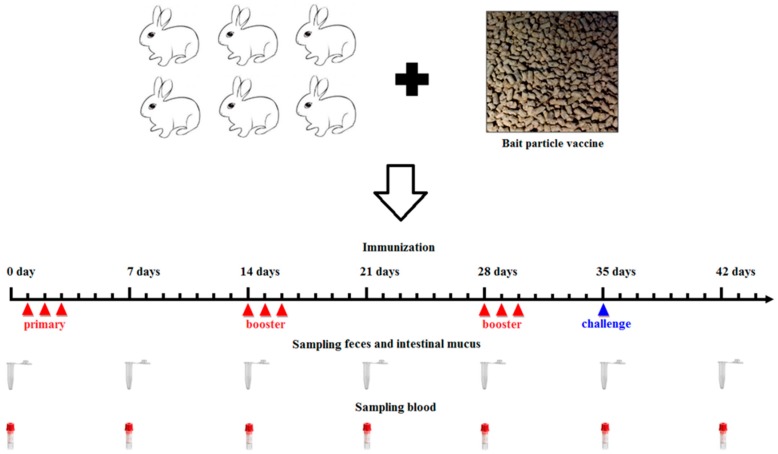
Diagrammatic representation of the oral immunization/challenge protocol and sampling time. Here, we used rabbits as an animal model to evaluate the immunogenicity of the bait particle vaccine. The immune protocol and sample collection times are shown in panel.

A bait particle vaccine was prepared with the pPG-α-β2-ε-β1/*L. casei* strain. Briefly, the overnight cultures of the pPG-α-β2-ε-β1/*L. casei* grown in MRS broth were thoroughly mixed with feed raw materials and cold-extruded into bait particles through an extruder type 210 with membrane hole of 6 mm (Jinquan Machinery Co., Ltd. Weihui, China) according to the manufacturer’s instructions. The bait particle vaccine contains, on average, approximately 10^10^ CFU/g of the pPG-α-β2-ε-β1/*L. casei*. The NZW rabbits in the probiotic vaccine group (*n* = 31) were fed bait particles containing the recombinant lactobacillus (Figure 1), and the control group (*n* = 31) was fed bait particles containing PBS or pPG-T7g10/*L. casei*. The immune protocol is shown in Figure 1 [23]. Animals were not fed for half a day before each immunization. In parallel, the group of NZW rabbits (*n* = 20) was injected intramuscularly with commercial inactivated *C*. *perfringens* vaccine made by *C*. *perfringens* toxinotypes A, C, and D (China Animal Husbandry Industry Co., Ltd. Beijing, China) according to the manufacturer’s instructions. This group was used as the vaccine control group to evaluate the immune protection effect of the recombinant lactobacillus pPG-α-β2-ε-β1/*L. casei*.

### 2.6. Detection of Antigen-Specific Antibodies and Cytokines

Serum, intestinal mucus, and fecal samples were collected from the NZW rabbits (*n* = 3) in each group on days 0, 7, 14, 21, 28, 35, and 42 after primary immunization and prepared as previously described, respectively [22]. Subsequently, antigen-specific sIgA levels in the intestinal mucus and fecal samples (diluted at 1:16) and antigen-specific IgG levels in serum samples (diluted at 1:32) were detected using ELISA against α, β1, β2, and ε toxoid proteins expressed by *E. coli* in our laboratory as the coat antigen and using HRP-conjugated goat anti-mouse IgA or IgG (Abcam, Cambridge, MA, USA) as the secondary antibody. In addition, IL-2, IL-4, IL-10, IL-12, and IFN-γ cytokine levels in the serum samples obtained from the NZW rabbits in each group were detected using ELISA kits (Biosource International, Waltham, MA, USA). Moreover, to determine the antibody levels of vaccine control group, the serum samples diluted at 1:500/1:10 used for detecting specific IgG/IgA antibody level and fecal samples (diluted at 1:1) used for detecting specific sIgA level were collected on day 0 and 35 after primary immunization before performing ELISA assay using inactivated *C*. *perfringens* vaccine antigens as the coating antigen.

### 2.7. Ability of Antibodies to Neutralize Exotoxins of C. perfringens

The exotoxin-neutralizing ability of IgG and sIgA antibodies obtained from the NZW rabbits orally immunized with the pPG-α-β2-ε-β1/*L. casei* was evaluated using the antibodies obtained from group pPG-T7g10/*L. casei* and PBS as a control. Briefly, undiluted IgG and sIgA antibodies collected from each group on day 35 after primary immunization were mixed with equal volume (1× lethal dose, LD_100_) of natural exotoxin mixtures produced by *C. perfringens* toxinotype A, C, and D, and incubated at 37 °C for 1 h. Next, eight-week-old healthy NZW rabbits (five rabbits for each group) were challenged with the exotoxin mixtures pretreated with the IgG/sIgA antibody via injection. The survival rate of rabbits in each group was determined.

### 2.8. Detection of CD4^+^ and CD8^+^ T Cells

Spleen lymphocytes of the NZW rabbits (*n* = 3) in each group were collected and adjusted to 2 × 10^6^/mL on day 35 after primary immunization. Subsequently, the lymphocytes were stained with FITC anti-rabbit CD4, APC anti-rabbit CD3, and PE anti-rabbit CD8 fluorescence antibody (Abcam, Cambridge, England) in dark conditions at 37 °C for 30 min, respectively. After washing three times with sterile PBS, flow cytometry analysis was performed using a FACS Caliber (Becton Dickinson, Franklin Lakes, NJ, USA).

### 2.9. Lymphoproliferation Activity Test

Briefly, the concentration of spleen lymphocytes obtained from the NZW rabbits (*n* = 3) in each group was adjusted to 5 × 10^6^ cells/mL, added to a 96-well plate (100 μL per cell in eight duplicates) containing RPMI 1640 plus 10% fetal bovine serum, and placed in a 5% CO_2_ incubator at 37 °C for 8–12 h. Subsequently, the cells were re-stimulated using purified α, β1, β2, and ε toxoid proteins at 0.01 mg/mL, 0.1 mg/mL, 1.0 mg/mL, or 10 mg/mL for 60 h, respectively, using RPMI 1640 culture medium as a blank control. Next, 5 mg/mL of thiazolyl blue tetrazolium bromide (MTT) was added into the cell plate (10 μL per well) and incubated at 37 °C for another 4 h followed by lymphoproliferation activity detection with CellTiter 96 AQueous Non-Radioactive Cell Proliferation Assay according to the manufacturer’s instructions (Promega, USA). The stimulation index was calculated as follows: SI = OD_600(sample)_/OD_600(blank control)_.

### 2.10. Challenge Tests

Briefly, on day 35 after the primary oral immunization, each rabbit (*n* = 10) in each group was intramuscularly injected with 1 × LD_100_ (90 mg of exotoxins mixture per kg body weight) of crude exotoxin mixture produced by *C. perfringens* toxinotype A, C, and D, and simultaneously orally challenged with 2 mL of *C. perfringens* toxinotype A, C, and D culture (the total concentration of culture mixture was 3 × 10^8^ CFU/mL, and 1 × 10^8^ CFU/mL for each toxinotype). The survival rate of rabbits in each group was recorded post-challenge, and histopathological changes in brain, heart, liver, spleen, lung, kidney, and intestine of rabbits in each group were observed.

### 2.11. Analysis of Intestinal Flora Diversity of Immunized Rabbits Challenged with C. perfringens

After challenging the rabbits with *C. perfringens*, we collected the fecal samples to analyze the changes in intestinal flora diversity of the immunized rabbits with the recombinant lactobacillus. Briefly, fecal samples were collected from the immunized rabbits (*n* = 3) on day 15 after oral challenge with *C. perfringens*. The Illumina MiSeq platform was used to perform 16S rRNA high throughout sequencing to analyze the intestinal flora diversity in fecal samples. In parallel, the fecal samples collected from rabbits in the PBS group and in the *C. perfringens* group were used as controls. Sequencing and data analysis was performed by Guangzhou Gene Denovo Biotechnology Co., Ltd., Guangzhou, China. The raw sequence reads were deposited into the NCBI database as a BioProject (accession number: PRJNA541228).

### 2.12. Statistical Analysis

GraphPad Prism V5.0 software (GraphPad Software, San Diego, CA, USA) was used to carry out statistical analyses for all data. The results are shown as means ± standard errors of five replicates per test in a single experiment repeated three times. Tukey’s multiple comparison tests and one-way analysis of variance (ANOVA) were used to analyze the significance of differences between groups. A *p* value < 0.05 (*) indicated statistical significance, and a *p* value < 0.01 (**) indicated highly significant differences.

## 3. Results

### 3.1. Constitutive Fusion Protein Expression

**Figure 2:**
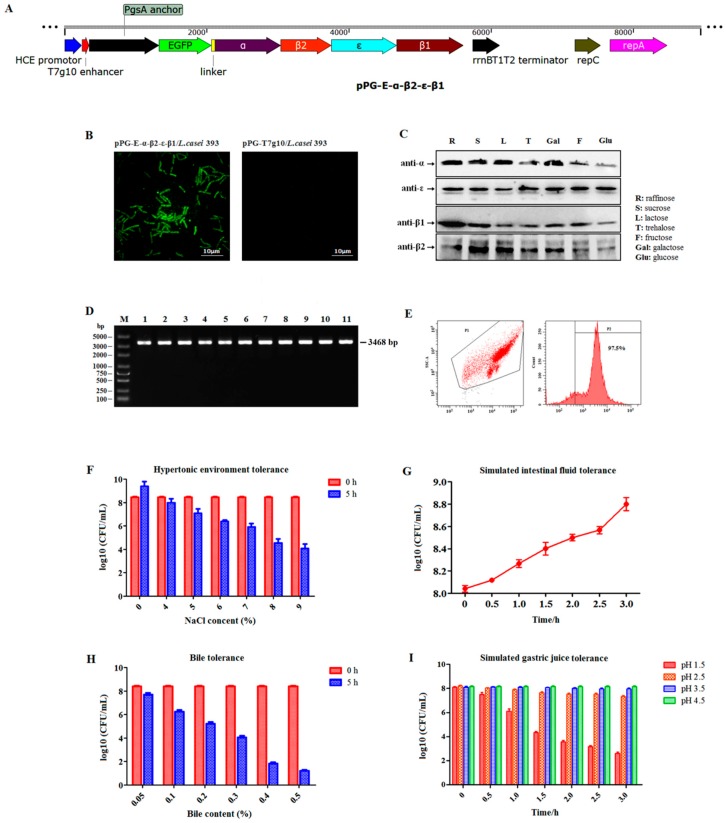
(**A**) Details of construction of the recombinant plasmid pPG-E-α-β2-ε-β1. (**B**) The constitutive expression of screening marker enhanced green fluorescent protein (EGFP) observed by ultra-high-resolution microscopy. (**C**) The constitutive expression of the fusion protein identified by western blotting using mouse anti-α, β2, ε, and β1 toxoid monoclonal antibody, respectively. (**D**) Segregational stability results of the recombinant lactobacillus with serial subcultures targeting the genes of interest. Lane 1: positive recombinant plasmid pPG-E-α-β2-ε-β1 control; lanes 2–11: the identification results of every five generations of subcultures pPG-E-α-β2-ε-β1/*L. casei* targeting the fusion gene α-β2-ε-β1 for 50 generations. (**E**) Segregational stability results of the recombinant lactobacillus targeting the screening marker EGFP. The tolerance of the recombinant lactobacillus to hypertonic environment (**F**), simulated intestinal fluid environment (**G**), 0.5% bile salt (**H**), and simulated gastric environment (**I**).

Here, we used EGFP as a marker gene to screen recombinant pPG-E-α-β2-ε-β1/*L. casei* 393 (Figure 2A). Therefore, the constitutive EGFP marker expression by the recombinant strain cultured in normal MRS medium without the presence of any inducers was first identified using ultra-high-resolution microscopy. The result showed that there was significant green fluorescence observed on the cell surface of pPG-E-α-β2-ε-β1/*L. casei*, but not on pPG-T7g10/*L. casei* (Figure 2B), indicating that EGFP was constitutively expressed. Subsequently, constitutive expression of the α-β2-ε-β1 fusion protein by recombinant pPG-E-α-β2-ε-β1/*L. casei* 393 was confirmed using western blotting. The results showed that the α-β2-ε-β1 fusion protein was successfully expressed by the pPG-E-α-β2-ε-β1/*L. casei* cultured without any inducers in basic MRS broth supplemented with different sugars as carbon sources. The α/β2/ε/β1 toxoid protein in the α-β2-ε-β1 fusion protein can be recognized using mouse anti-α, β2, ε, and β1 toxoid monoclonal antibodies, respectively (Figure 2C).

### 3.2. Segregational Stability of Recombinant Lactobacillus

The results indicated that the recombinant plasmid was stable with no loss of the target genes (Figure 2D) or any structural rearrangement (sequencing data not shown). After 50 generations of subcultures, the segregational stability was also determined using flow cytometry targeting the EGFP marker. The rate of EGFP-positive recombinant lactobacillus was as high as 97.5% (Figure 2E), indicating good segregational stability of the pPG-E-α-β2-ε-β1/*L. casei* strain.

### 3.3. Digestive Environment Tolerance in Recombinant Lactobacillus

The tolerance of recombinant pPG-E-α-β2-ε-β1/*L. casei* to bile, hypertonic environment, simulated gastric environment, and intestinal fluid environment was evaluated. Results showed that in extreme environments—hypertonic environment (9% NaCl for 5 h), 0.5% bile salt for 5 h, and simulated gastric environment (pH 1.5) for 3 h—the survival rate of the pPG-E-α-β2-ε-β1/*L.casei* was still maintained at approximately 50% (Figure 2F), 25% (Figure 2H), and 25% (Figure 2I), respectively. The results indicated that the recombinant lactobacillus had a certain level of tolerance to extreme digestive environments. Remarkably, the recombinant lactobacillus showed growth tendency in the simulated intestinal fluid environment (Figure 2G).

### 3.4. Recombinant Lactobacillus Colonization in Rabbit Intestinal Tracts

**Figure 3:**
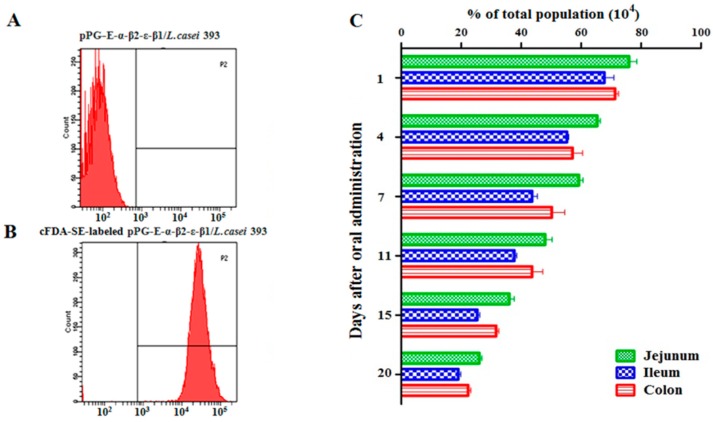
Colonization ability in the intestine of the recombinant lactobacillus. (**A**,**B**) Unlabeled and cFDA-SE-labeled pPG-E-α-β2-ε-β1/*L. casei* analyzed using flow cytometry. (**C**) Colonization of the recombinant lactobacillus in the intestinal tracts of rabbits. Results showed that the colonization rate of the recombinant lactobacillus gradually decreased, while the positive rate of the cFDA-SE-labeled lactobacillus still maintained 20–25% on day 20 after oral administration.

The adhesion ability of pPG-E-α-β2-ε-β1/*L. casei* in the intestinal tracts of rabbits was evaluated using flow cytometry after oral administration of the cFDA-SE-labeled recombinant lactobacillus (unlabeled bacteria as shown in Figure 3A, cFDA-SE-labeled bacteria as shown in Figure 3B). As shown in Figure 3C, the colonization rates of cFDA-SE-labeled lactobacillus in the jejunum, ileum, and colon on day 1 after oral administration were approximately 75%, 63%, and 66% respectively, compared to the 5–8% background (negative control group) fluorescence value that has been subtracted from the final results. Although the colonization rate of the pPG-E-α-β2-ε-β1/*L. casei* gradually decreased, the rate of cFDA-SE-labeled lactobacilli was still maintained at 20–25% in the intestinal tracts of rabbits, indicating good adhesion ability.

### 3.5. Determination of Antigen-Specific Antibody Levels after Oral Immunization

**Figure 4:**
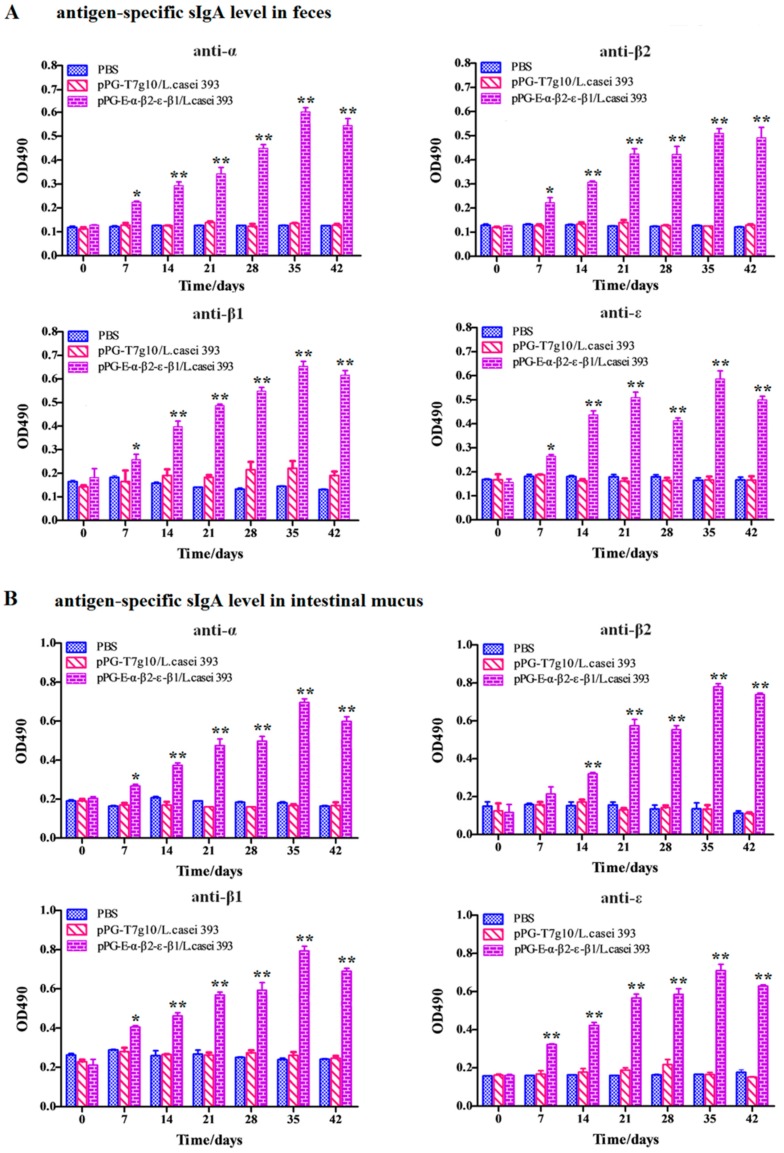
The levels of antigen-specific sIgA antibody in the feces (**A**) and in the intestinal mucus (**B**) of rabbits were determined using ELISA at different time points after the primary immunization with the bait particle vaccine. The results are presented as mean ± SD of each group (* *p* < 0.05, ** *p* < 0.01).

**Figure 5:**
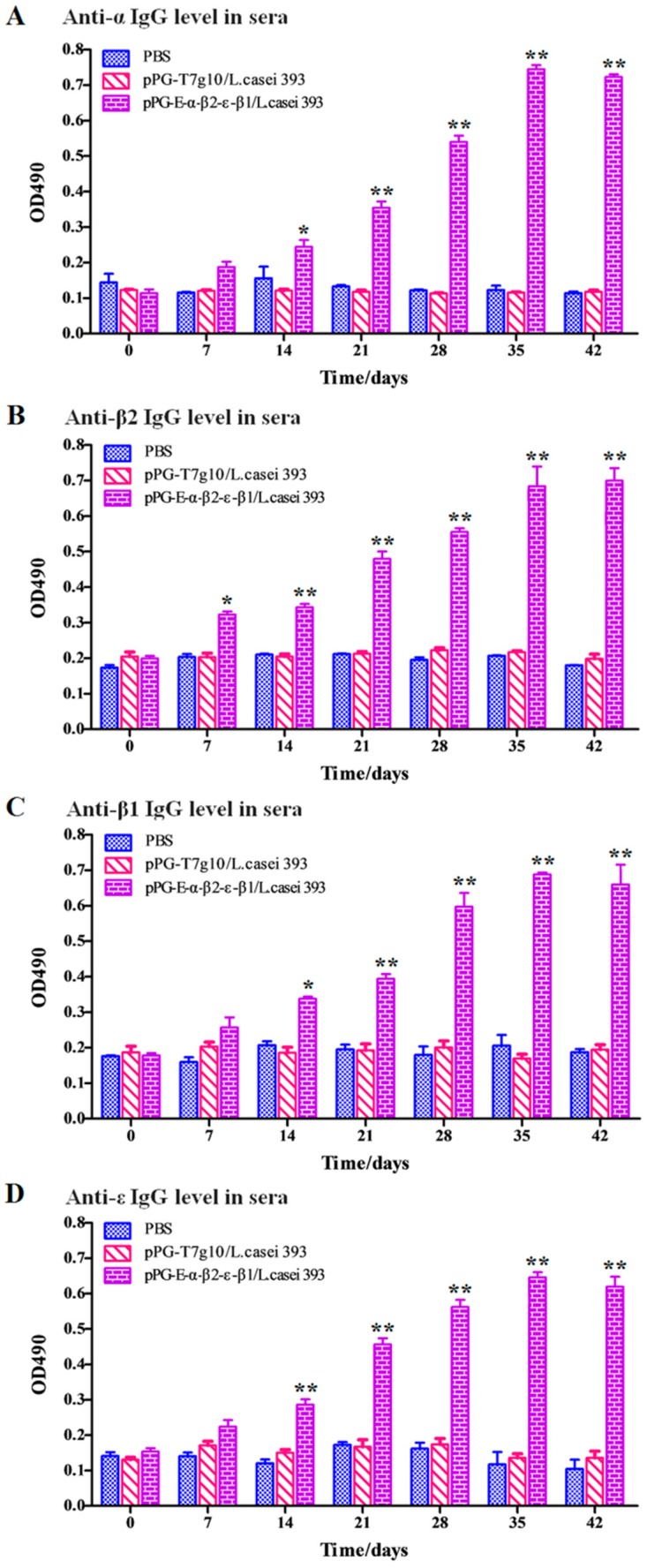
The levels of antigen-specific IgG antibody in serum samples of rabbits were determined using ELISA at different time points after the primary immunization with the bait particle vaccine. (**A**) Anti-α IgG level in sera; (**B**) Anti-β2 IgG level in sera; (**C**) Anti-β1 IgG level in sera; (**D**) Anti-ε IgG level in sera. The results are presented as mean ± SD in each group (* *p* < 0.05, ** *p* < 0.01).

**Figure 6:**
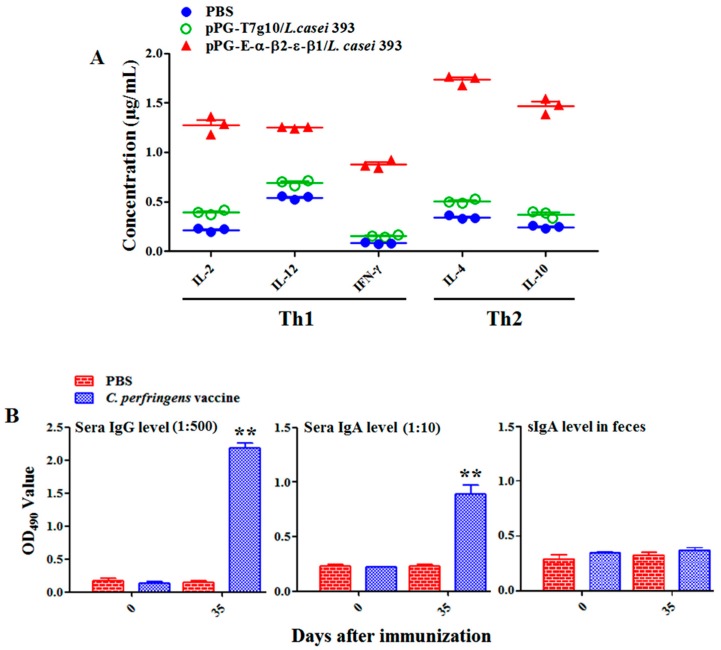
The levels of cytokines IL-2, IL-4, IL-10, IL-12, and IFN-γ in serum samples were determined using ELISA (**A**), and the antibody levels in the rabbits immunized by commercial vaccine via injection (**B**). The results are presented as mean ± SD of each group (* *p* < 0.05, ** *p* < 0.01).

Oral immunization with the bait vaccine effectively induced antigen-specific sIgA-based mucosal (Figure 4) and IgG-based systemic immune responses (Figure 5), indicating good oral immunogenicity. Significant levels (*p* < 0.01) of antigen-specific sIgA in feces (Figure 4A), in intestinal mucus (Figure 4B), and antigen-specific IgG in sera (Figure 5) were elicited in the immunized rabbits on days 14 and 21 after oral immunization compared to group pPG-T7g10/*L. casei* and PBS (no difference in the levels of antigen-specific sIgA and IgG antibodies before and after oral immunization, *p* > 0.05). Significantly, the antigen-specific sIgA and IgG antibodies induced by the recombinant lactobacillus effectively neutralized the main exotoxins of *C. perfringens* toxinotypes A, C, and D, and they provided effective protection against exotoxins (Table 1). At the same time, significant levels (*p* < 0.01) of Th1-associated cytokines IL-2, IL-12, and IFN-γ and Th2-associated cytokines IL-4 and IL-10 were determined in the serum samples collected from the immunized rabbits with the bait vaccine compared to group pPG-T7g10/*L. casei* or PBS (Figure 6A). In addition, we determined the antibody levels induced by the commercial inactivated *C*. *perfringens* vaccines. Significant levels of serum IgG and serum IgA antibodies were elicited after immunization (*p* < 0.01) (Figure 6B). However, there was no significant difference (*p* > 0.05) in the level of specific intestine mucosal sIgA antibody before and after immunization.

### 3.6. CD4^+^ and CD8^+^ T Cell Assay

**Figure 7:**
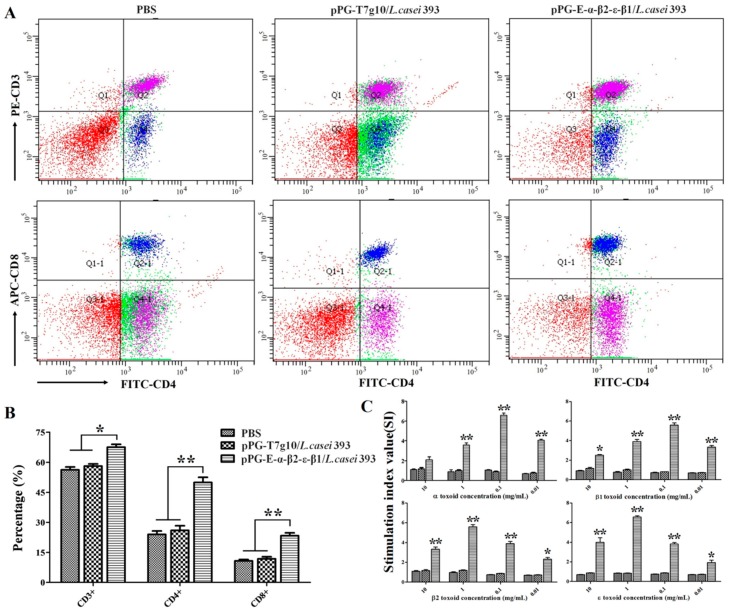
The percentage of CD4^+^ and CD8^+^ T cells in rabbit splenic lymphocytes in each group was determined using flow cytometry on day 35 after primary immunization (**A**). Data are presented as mean ± SD in each group (**B**). Moreover, lymphoproliferation was tested after re-stimulation with purified α, β1, β2, and ε toxoid proteins, and the results are presented as mean ± SD of each group (* *p* < 0.05, ** *p* < 0.01) (**C**).

The splenic lymphocytes were collected from the rabbits in each group on day 35 after primary immunization, followed by the determination of the percentage of CD3^+^, CD4^+^, and CD8^+^ T cells using flow cytometry (Figure 7A). The results showed that the percentages of CD3^+^, CD4^+^, and CD8^+^ T cells in the splenic lymphocytes of the immunized rabbits with the bait vaccine were remarkably increased (*p* < 0.01) compared to the pPG-T7g10/*L. casei* group or PBS group (there were no significant differences observed) (Figure 7B).

### 3.7. Lymphoproliferation Assay

To analyze the ability of the bait particle vaccine to promote lymphocyte proliferation, the rabbit splenic lymphocytes in each group were collected on day 35 after the primary immunization and re-stimulated using purified α, β1, β2, or ε toxoid proteins. Remarkable lymphoproliferation (*p* < 0.01) was observed in the rabbits that were orally immunized with the bait particle vaccine containing pPG-E-α-β2-ε-β1/*L. casei* compared to the pPG-T7g10/*L. casei* group or PBS group (Figure 7C).

### 3.8. Immune Protection Effect of the Bait Particle Vaccine against C. perfringens Toxins

The challenge test showed that the survival rate in the rabbits orally immunized with the bait particle vaccine was 80%, and it was 60% in rabbits intramuscularly injected with the commercial inactivated *C*. *perfringens* vaccine. However, all of the rabbits in the pPG-T7g10/*L. casei* group and PBS group died (Table 1). Our results indicated that the bait particle vaccine was more efficacious in providing immune protection for the rabbits against *C. perfringens* exotoxins.

**Figure 8:**
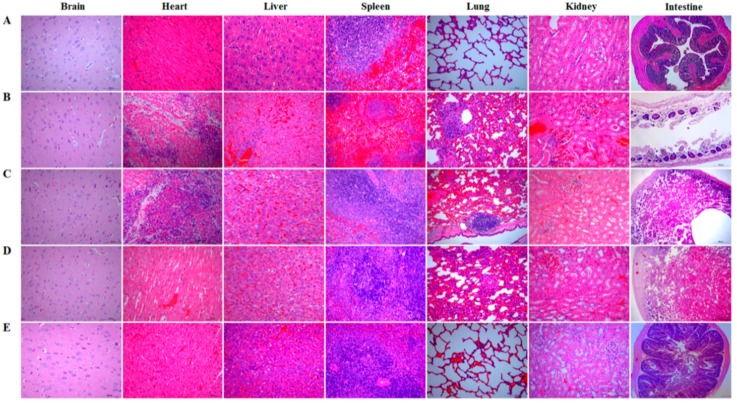
Histopathological changes in the brain, heart, liver, spleen, lung, kidney, and intestine tissues of the rabbits in each group were observed after intramuscular injection with 1 × LD_100_ of crude exotoxin mixture produced by *C. perfringens* toxinotype A, C, and D combined with oral administration of *C. perfringens* toxinotype A, C, and D culture. Sections were stained with hematoxylin and eosin and photographed at 40× magnification. (**A**) normal group; (**B**) PBS group; (**C**) pPG-T7g10/*L. casei* group; (**D**) commercial vaccine group; (**E**) bait particle vaccine group.

After the challenge test, the brain, heart, liver, spleen, lung, kidney, and intestinal tissues of the rabbits in each group were isolated and subjected to histopathological observation. No abnormal histopathological changes were observed in the brain, heart, spleen, lung, and kidney tissues of the rabbits in the bait particle vaccine group, and mild hemorrhage in the liver and slight disruption of the intestinal villi were observed (Figure 8E) compared to histopathological changes in the tissues of the rabbits in the normal group (Figure 8A). There were also no abnormal histopathological changes observed in the brain, heart, spleen, and kidney tissues of rabbits in the commercial vaccine group. However, mild hemorrhage in the liver, moderate widening of the pulmonary-alveolar diaphragm, particularly complete disruption of intestinal mucosa structure, and intestinal epithelial necrosis were observed (Figure 8D). Remarkable abnormal histopathological changes were observed in rabbits in pPG-T7g10/*L. casei* group (Figure 8C) and PBS group (Figure 8B), including myocardial cell degeneration and necrosis, lymphocyte and neutrophil infiltration in the heart, severe widening of the pulmonary-alveolar diaphragm, focal aggregation of lymphocytes near lung bronchioles, severe disruption of intestinal structural integrity, and intestinal epithelial necrosis in intestine. In addition, slight congestion and hemorrhage changes were found in liver and kidney tissues of the rabbits in the pPG-T7g10/*L. casei* and PBS groups, and spleen red pulp of the rabbits dilated significantly in the PBS group.

### 3.9. Intestinal Flora Diversity of Immunized Rabbits after Challenge

**Figure 9:**
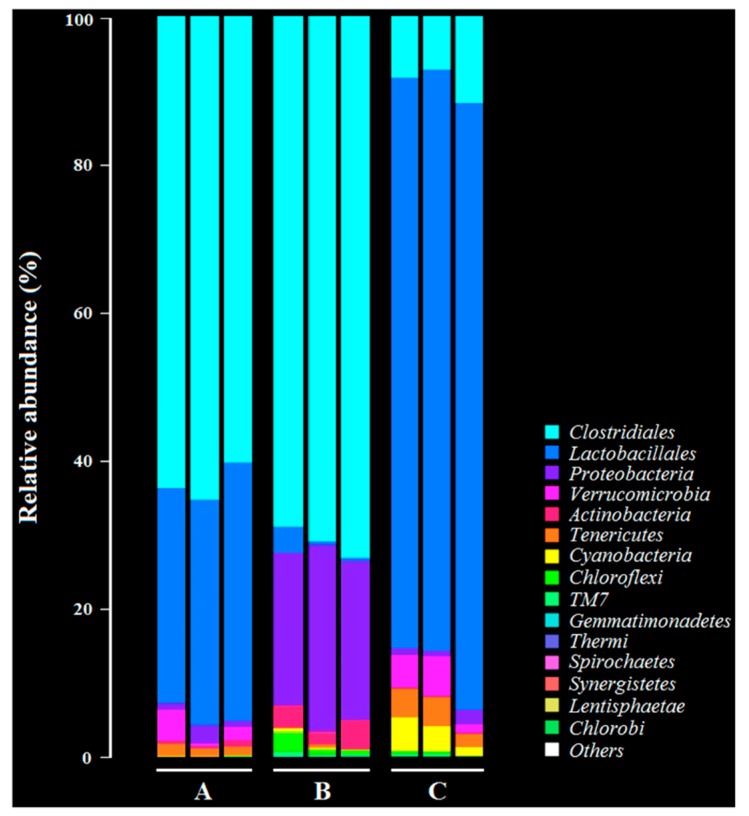
Intestinal flora diversity of the immunized rabbits after challenge with *C. perfringens* determined by the Illumina MiSeq high throughput sequencing platform targeting 16S rRNA. (**A**) Intestinal flora diversity in normal rabbits. (**B**) Intestinal flora diversity in rabbits challenged with *C. perfringens*. (**C**) Intestinal flora diversity in immunized rabbits with bait particle vaccine after challenge with *C. perfringens*.

As shown in Figure 9, the most significant changes in the intestinal flora diversity of the rabbits were observed in the members of *Clostridiales*, *Lactobacillales*, and *Proteobacteria* before and after oral immunization with the bait particle vaccine. In the normal group, the dominant bacterial flora were mainly from *Clostridiales* and *Lactobacillales* (Figure 9A). The number of bacteria from *Lactobacillales* decreased remarkably (*p* < 0.01) after *C. perfringens* challenge, but the number of bacteria from *Proteobacteria* increased remarkably (*p* < 0.01) (Figure 9B) compared to group A. Significantly, the most dominant bacterial flora in the intestinal tract of the immunized rabbits with the bait particle vaccine after challenge by *C. perfringens* was still from *Lactobacillales* (*p* < 0.01). However, the number of bacteria from *Clostridiales* and *Proteobacteria* significantly decreased (*p* < 0.01) (Figure 9C) compared to group B.

## 4. Discussion

*C. perfringens* is an opportunistic pathogen commonly found in animal intestines. Exotoxins are the main virulence factors associated with *C. perfringens* lethality, cytotoxicity, necrosis, and hemorrhagic activity. These exotoxins are mainly absorbed through the intestinal tracts of animals, resulting in enterotoxemia, dysentery, and hemorrhagic enteritis [5,25]. Although available commercial intramuscular injection vaccines (inactivated bacterins or subunit vaccines) have been used to prevent *C. perfringens*-mediated diseases in livestock, these vaccines cannot effectively inhibit the invasion of pathogenic bacteria through intestinal tracts or toxin absorption through intestinal mucosa. Therefore, developing a vaccine that could effectively induce both antigen-specific intestinal mucosal and systemic immune responses against exotoxins is very important for preventing and controlling *C. perfringens*-mediated diseases. An oral vaccine may be a promising strategy [26].

In our previous study, we constructed a recombinant lactobacillus pPG-E-α-β2-ε-β1/*L. casei* expressing tetravalent *C. perfringens* toxoids. This showed a good immunogenicity in mice after oral administration [24]. Here, a rabbit model was used to explore the immunogenicity and immune protection effect of the recombinant lactobacillus in livestock. Before immunization, we evaluated its segregational stability. Our data indicated that the recombinant lactobacillus had good stability properties with no target gene loss or structural rearrangement. Subsequently, we systemically evaluated the tolerance of the recombinant lactobacillus to digestive environments. Although there was an approximately 75% drop in recombinant lactobacillus in 0.5% bile salt conditions for 5 h and a gastric environment of pH 1.5 for 3 h, we found that the survival rate was efficient for the activity of the vaccine, which was confirmed by subsequent immunization experiment. In addition, in order to further enhance the immune effect of the recombinant lactobacillus, the oral immunization protocol was performed on three consecutive days (days 0, 1, and 2), and a booster immunization was given at days 14, 15, and 16, and a second booster was given at days 28, 29, and 30. The adhesion ability in the intestinal tracts is also important and desirable for live oral probiotic vaccine. After evaluating this characteristic, we found that the recombinant lactobacillus had good adhesion and colonization ability in the intestinal tracts of rabbits. In addition, we prepared the bait particle vaccine as a mixture of recombinant lactobacillus and feed raw materials to facilitate immunization via oral route; this reduced the workload and prevented injection stress.

As an oral vaccine, it is generally expected to efficiently induce both antigen-specific intestinal mucosal and systemic immune responses. Therefore, we evaluated the immunogenicity of the bait particle vaccine in rabbits via oral administration. Secretory IgA (sIgA) is the predominant antibody at the mucosal surface, and it plays a key role in host defense against mucosal infections [27]. Here, we found that a significant level (*p* < 0.05) of antigen-specific sIgA was elicited in the rabbits orally immunized with recombinant lactobacillus from the 7th day onwards, and an extremely significant level (*p* < 0.01) was elicited from the 14th day onwards after the primary immunization. Moreover, a significant level (*p* < 0.01) of antigen-specific IgG was elicited from the 21st day onward after the primary immunization. Along with the results obtained from our previous study using mice as animal model [24], we can confirm that the recombinant lactobacillus can effectively induce antigen-specific intestinal mucosal and systemic immune responses after oral administration. Significantly, the antigen-specific sIgA and IgG antibodies that were induced by the bait vaccine showed effective *C. perfringens* exotoxin-neutralizing activity that contributed to host immune defense by preventing the absorption of exotoxins in the gut and their entrance into the systemic circulation. Although modified lactobacillus has been used to express *C. perfringens* α toxoid [23] and ε toxoid [28] for oral vaccine development, it cannot provide effective protection for the animals against other major exotoxins, especially against multiple α, β, and ε exotoxins at the same time.

Generally, lymphoproliferation positively correlates with cellular immunity. Our results indicated that the bait vaccine could induce cellular immunity. Significant levels (*p* < 0.01) of Th1-type (IL-2, IL-12, IFN-γ) and Th2-type (IL-4, IL-10) cytokines were detected in the sera of the rabbits immunized with the bait vaccine compared to control groups, indicating induction of mixed Th1- and Th2-type immune responses by the bait vaccine. We also determined the levels of CD4^+^ and CD8^+^ T cells, finding that the percentages of CD4^+^ and CD8^+^ T cells in splenocytes of the rabbits immunized with the bait vaccine increased significantly and indicating that vaccination with the bait vaccine could promote proliferation of CD4^+^ and CD8^+^ T lymphocytes.

A vaccine against *C*. *perfringens*-mediated diseases should provide effective immune protection against *C. perfringens* exotoxins in animals. Therefore, we evaluated the protective effect of the bait vaccine after a challenge model of exotoxins plus pathogens using a commercial inactivated *C*. *perfringens* vaccine as a vaccine control. The protection rates for the rabbits immunized with the bait vaccine and commercial vaccine were 80% and 60%, respectively, compared to group pPG-T7g10/*L. casei* or PBS (all rabbits died). Significantly, the protection effect provided by the bait vaccine was better than that provided by the commercial vaccine. Furthermore, the most significant histopathological changes between the groups were observed in the lung and intestine tissues. The lung and intestine tissues in the commercial vaccine group developed severe clinical symptoms, particularly the intestinal tissue which developed enteritis necroticans. This indicated that the commercial vaccine administered via injection could not provide effective protection for the intestine tissue against *C. perfringens* exotoxins, but the bait vaccine could. This possibly explains the lower protection rate of the commercial vaccine compared to the bait vaccine. Therefore, antigen-specific sIgA and IgG antibodies elicited by the bait vaccine contributed to the host immune defense by preventing diseases induced by exotoxin absorption in the gut and entrance into the systemic circulation. Moreover, we found that *L. casei* 393 had good probiotic properties, which could promote the elimination of pathogenic bacteria from the intestinal tracts, enhance animal feed intake and daily gain, increase feed utilization rate, and reduce the feed-to-weight ratio (data not shown).

## 5. Conclusions

In summary, the oral bait particle vaccine prepared with the recombinant lactobacillus constitutively expressing α, ε, β1, and β2 toxoids effectively induced potent antigen-specific mucosal and humoral immune responses and provided effective protection for animals against *C. perfringens* exotoxins. Significantly, the bait probiotic vaccine facilitated immunization, reduced workload, and prevented injection stress, suggesting a promising vaccine against *C. perfringens*-mediated diseases.

## Figures and Tables

**Table 1:** Immune protection efficacy of the bait particle vaccine.

	Groups	Dose	Survival
IgG antibody-neutralizing activity	pPG-E-α-β2-ε-β1/*L. casei* 393	IgG ^1^ + 1 × LD_100_ toxins mixture ^2^	5/5
	pPG-T7g10/*L. casei* 393		0/5
	Commercialized vaccine		5/5
	PBS		0/5
sIgA antibody-neutralizing activity	pPG-E-α-β2-ε-β1/*L. casei* 393	sIgA ^3^ + 1 × LD_100_ toxins mixture	5/5
	pPG-T7g10/*L. casei* 393		0/5
	Commercialized vaccine		0/5
	PBS		0/5
Challenge post-immunization	pPG-E-α-β2-ε-β1/*L. casei* 393	1 × LD_100_ toxins mixture + *C. perfringens* toxinotype A, C, and D culture ^4^	8/10 ^a^
	pPG-T7g10/*L. casei* 393		0/10 ^c^
	Commercialized vaccine		6/10 ^b^
	PBS		0/10 ^c^

^1.^ IgG antibody was obtained from the rabbits in each group. ^2.^ 1 × LD_100_ (90 mg exotoxins mixture per kg body weight) of crude exotoxins produced by *C. perfringens* toxinotype A, C, and D. ^3.^ sIgA antibody was obtained from the rabbits in each group. ^4.^ The total concentration of culture mixture of *C. perfringens* toxinotype A, C, and D was 3 × 10^8^ CFU/mL (1 × 10^8^ CFU/mL for each toxinotype). The letters (a vs. c, b vs. c) indicate extremely significant differences (*p* < 0.01) of protection rates; the letters (a vs. b) indicate significant differences (*p* < 0.05) of protection rates.
